# *Lactobacillus plantarum* strain HT-W104-B1: potential bacterium isolated from Malaysian fermented foods for control of the dermatophyte *Trichophyton rubrum*

**DOI:** 10.1007/s11274-021-03020-7

**Published:** 2021-02-24

**Authors:** Azlina Mohd Danial, Angel Medina, Naresh Magan

**Affiliations:** 1 Applied Mycology Group, Environment and AgriFood Theme, Cranfield University, Cranfield, Bedford, MK43 0AL UK; 2 Present Address: Science and Food Technology Research Centre, Malaysian Agricultural and Research Institute, 43400 Serdang, Selangor Malaysia

**Keywords:** Anti-fungals, Dermatophyte, Lactic acid bacteria, Metabolites, Organic acids

## Abstract

The objective was to screen and evaluate the anti-fungal activity of lactic acid bacteria (LABs) isolated from Malaysian fermented foods against two *Trichophyton* species. A total of 66 LAB strains were screened using dual culture assays. This showed that four LAB strains were very effective in inhibiting growth of *T. rubrum* but not *T. interdigitale.* More detailed studies with *Lactobacillus plantarum* strain HT-W104-B1 showed that the supernatant was mainly responsible for inhibiting the growth of *T. rubrum*. The minimum inhibitory concentration (MIC), inhibitory concentration, the 50% growth inhibition (IC_50_) and minimum fungicide concentration (MFC) were 20 mg/mL, 14 mg/mL and 30 mg/mL, respectively. A total of six metabolites were found in the supernatant, with the two major metabolites being L-lactic acid (19.1 mg/g cell dry weight (CDW)) and acetic acid (2.2 mg/g CDW). A comparative study on keratin agar media showed that the natural mixture in the supernatants predominantly contained L-lactic and acetic acid, and this significantly controlled the growth of *T. rubrum*. The pure two individual compounds were less effective. Potential exists for application of the natural mixture of compounds for the treatment of skin infection by *T. rubrum*.

## Introduction

Malaysia has a wide range of traditional or naturally fermented foods made from plant and animal sources. Among these are *tapai* (fermented tapioca, fermented glutinous rice),* tempoyak* (fermented durian flesh), *budu* (fermented fish sauce), *pekasam* (fermented fish), *belachan* (fermented shrimps paste) and fermented vegetables. For fermented tapioca and glutinous rice, these are boiled or steamed, mixed with starter cultures, wrapped in banana or rubber plant leaves and kept at room temperature for 2 days. For other fermented food products, this involves the use of salt and/or rice and water that are mixed together with the food and kept at room temperature in a closed environment for several weeks or months. Numerous microorganisms have been isolated from these indigenous Malaysian fermented foods (Moreno et al. 2002; Mohd Adnan and Tan 2007; Zareian et al. 2012). Of particular interest are the LABs which are known to produce a range of potentially useful anti-microbial compounds (Jang et al. 2014; Lay et al. 2016; Schmidt et al. 2018). There has been interest in screening these LABs for efficacy against other important food and medicial fungal pathogens.

Dermatophytes are a group of pathogenic fungi that predominantly cause superficial skin diseases on humans and other mammals (Teklebirhan and Bitew 2015; Kim et al. 2016; AL-Khikani 2020). Indeed, all age groups can be affected, regardless of whether the patients are immunocompromised or not (Thomas et al. 2010; Wu et al. 2013; Farag et al. 2018). They need a keratin rich surface to grow on and thus predominantly infect skin, nails and hair of humans and animals. In addition, there are reports of deep dermatophytosis in immunosuppressed patients although these cases are relatively uncommon (Hainer 2003; Gong et al. 2007). The most common dermatophyte fungal species include *Trichophyton, Microsporum* and *Epidermophyton* genera. These dermatophytes can be transmitted from human to human (antrophophilic organisms), soil to humans on contact (geophilic organisms) or via animals to humans (zoophilic organisms) (Hainer, 2003; AL-Khikani 2020). This is not a life-threatening disease, but the prevalence of people infected with dermatophytic fungi has been continuously increasing in many countries (Vena et al. 2012; Kim et al. 2016; Farag et al. 2018; Singla et al. 2019; Van et al. 2019).

In addition, the susceptibility of dermatophytes to anti-fungal drugs has continuously decreased. Ghannoum (2016) reported the resistance of dermatophytes to azoles had increased to 19% among the worldwide population. Earlier, Ghelardi et al. (2014) and Mukherjee et al. (2003) revealed that the resistance of *T. rubrum* isolated from patients to anti-fungal drugs had increased after prolong exposure to sub-inhibitory concentration of itraconazole, amorolfine and terbinafine. In addition to this, the terbinafine resistant *T. rubrum* strains also showed cross-resistance to naftifine, butenafine, tolnaftate and tolciclate (Mukherjee et al. 2003; Sinha and Sardana, 2018). This is a serious problem, as these drugs are used clinically either for oral (terbinafine and itraconazole) or topical treatment for dermatophyte infections and suggests the need for new treatments (Scorzoni et al. 2017).

Furthermore, the patients with oral drug therapy are prone to the risk of drug-drug interactions. This is more severe particularly for immunocompromised patients including those with diabetes, liver disease, renal dysfunctions and the elderly (Gupta et al. 2013; AL-Khikani 2020). Therefore, the application of natural anti-fungal compounds, which are generally less toxic or non-toxic to patients, for both normal and immunocompromised patients would be very beneficial.

Many studies have been carried out to try and identify alternative treatments, especially from natural sources. Although many plant extracts with anti-dermatophyte activities have been reported (Koroishi et al. 2008; Khan and Ahmed 2011; Li et al. 2015), no clinical studies have been carried out to validate their efficacy. The use of alternative antioxidants has also been compared with itraconazole and griseofulvin efficacy to identify alternative control compounds using volatile production patterns (Naraghi et al. 2010). However, the consistency and potential for use on patients has not been evaluated.

The potential use of microorganisms, particularly LABs, could be one promising approach to overcome the problem of the build-up of resistance in dermatophytic fungi to existing anti-fungal drugs. There has been interest in the utilisation of these beneficial microorganisms or their metabolites as control compounds for human microbial pathogens. Very few, if any LABs isolated from a range of Malaysian fermented foods, have been previously screened for efficacy and control of dermatophytes such as *T. rubrum.*

The objectives of this study were to (a) screen 66 LABs for the control of a *T. rubrum* strain, (b) identify the best LABs which can control growth of this pathogen, (c) identify the dominant compounds produced by the best identified LAB and (d) quantify the MIC and IC_50_ concentrations necessary for inhibiting growth of this important dermatophyte.

## Materials and methods

### Preparation of fungal spore suspensions

Pathogenic strains of *Trichophyton rubrum* (N115) and *T. interdigitale* (N223) were obtained from the Royal Gloucester Hospital Trust (U.K.). The strains were grown on Sabouraud Dextrose Agar (SDA; Oxoid Ltd) at 30 °C for up to 14 days to ensure spore production. The spores were harvested using sterile 0.1% Tween-80/water solution and gently scraping the colony surface with a surface sterilised glass rod. The spore suspensions were decanted into sterile 50 ml tubes, centrifuged at 2000×*g* for 2 min and the supernatants were discarded. A sterile 10 mL aliquot of 0.1% Tween 80/water solution was added and the spore suspension thoroughly mixed and the concentration determined using a haemocytometer (Thoma, Germany). This was adjusted by the addition of sterile 0.1% Tween 80/water solution to obtain 2–5 × 10^6^ spores/mL.

### Preparation of LAB cell suspensions and dried cell free supernatants

The LABs screened consisted of: *Lactobacillus* (34 strains), *Lactococcus* (7 strains), *Leuconostoc* (4 strains) and *Pediococcus* (21 strains). These were isolated from a range of Malaysian fermented foods (Mohd Danial 2019). Each LAB strain in glycerol stock solution was inoculated onto sterile de Man, Rogosa, Sharpe (MRS; Oxoid Ltd) agar and incubated at 30 °C for 20 h. The cells were harvested by gently scraping using a sterile spreader into sterile sodium chloride (NaCl 0.85%; w/v) solution and transferred aseptically into sterile 50 mL conical tubes. The cell suspensions were then centrifuged at 2000×*g* at 4 °C for 2 min. The NaCl solution was discarded and fresh sterile 0.85% NaCl solution was added, and the density adjusted to approximately 1.0 at OD_600_ which was approx. 5 × 10^7^ CFUs/mL. The cell suspensions (300 µL) were inoculated into sterile 30 mL of MRS broth and incubated at 30 °C for 48 h in static conditions. The cell free supernatants were obtained by centrifugation at 2000×*g* for 10 min at 4 °C. This was then filter sterilized using a sterile cellulose acetate membrane filter (0.22 µm, Sartorius, Göttingen, Germany) and freeze dried aseptically. The dried cell free supernatants (CFS) were dissolved in sterile distilled water for the screening assays against *T. rubrum*.

### Screening for bacterial antagonists against *Trichophyton* using dual culture assays

A single colony of each LAB was streak inoculated on MRSA as a 2 cm line approx. 2 cm from the 9 cm Petri plate edge. After incubation at 30 °C overnight 5 µL of a spore suspension (10^6^ spores/mL) of the *T. rubrum* and *T. interdigitale* was applied (Sultan and Magan 2011) at a distance of 3–4 cm from the LABs and incubated at 30 °C for 20 days. The inhibition achieved was quantified using the fungal colony area and making comparisons with the control containing no LABs. The macroscopic interaction between the dual cultures with each colony was given an individual numerical score and compared. These were added together to obtain an overall Index of Dominance (I_D_) as developed by Magan and Lacey (1984). Each interacting species was given an individual score based on the following numerical values: 1:1-mutual intermingling, 2:2-mutual antagonism on contact, 3:3-mutual antagonism at a distance, 4:0-dominance of the one species on contact and 5:0-dominance of one species over the other at a distance.

### Antifungal efficacy of LABs grown in modified MRS (mMRS) broth

*Lactobacillus plantarum* strain HT-W104-B1 (*Lp* HT-W104-B1), *L. plantarum* strain MCC 2156 (*Lp* MCC 2156), *Pediococcus acidilactici* strain 1498 (*Pa* 1498), *P. pentosaceus* strain 1426 (*Pp* 1426) and a mixture of all four cell suspensions were inoculated into 40 mL sterile mMRS broth (mycological peptone 10 g/L, ‘Lab-Lamco’ powder 8 g/L, yeast extract 4 g/L, glucose 20 g/L, dipotassium hydrogen phosphate 2 g/L, magnesium sulphate 0.2 g/L and manganese sulphate 0.05 g/L; pH 6.2) and incubated at 30 °C for 48 h in static conditions. Periodically, after 24 and 48 h, 20 mL of the culture was removed and the supernatant separated from the cells by centrifugation at 2000×*g* at 4 °C for 10 min. This was then filter sterilized using a sterile cellulose acetate membrane filter (0.22 µm) and freeze dried aseptically. The dried cell free supernatants (CFS) were dissolved in sterile distilled water to a 20-fold concentration. The antifungal activity against *T. rubrum* was measured using the agar spot assay. Different combinations of *Lp* HT-W104-B1 and *Lp* MCC 2156 were also used to examine the effect of single and co-cultures on efficacy against *T. rubrum*.

### Effect of heat and pH modification of the supernatant from *Lp *HT-W104-B1 on control of *T. rubrum* growth

The nature of the anti-fungal compounds produced by LAB *Lp HT-W104-B1* strain were examined. The treatments were: (1) the supernatant at the original pH (3.3) was autoclaved at 121 °C for 15 min and (2) the pH was adjusted to 6.8 and then autoclaved at 121 °C for 15 min. The supernatant was freeze dried and later dissolved in sterile distilled water to a 15-fold concentration. The efficacy against *T. rubrum* was examined using the agar spot assay.

### Determination of the MIC, IC_50_ and MFC of the ***Lp*** HT-W104-B1 strain supernatant

Different concentrations of the supernatant (0–30 mg/mL) were evaluated to determine the MIC, IC_50_ and minimum fungicide concentration (MFC) against *T. rubrum* growth on keratin agar using the agar dilution method (EUCAST 2000). Keratin agar media consists of 0.5% keratin (w/v) (Santa Cruz Biotechnology Inc, Texas, USA), MgSO_4_.H_2_O (0.5 g/L), KH_2_PO_4_ (0.1 g/L), FeSO_4_.7H_2_O (0.01 g/L) and Zn SO_4_.7H_2_O (0.005 g/L), with a pH of 4.5 (Wawrzkiewicz et al. 1991).

### Identification of bioactive compounds from *Lp* HT-W104-B1 supernatant

Sample preparation, detection and quantification were performed using the multi-metabolite method developed by Malachová et al. (2014). Briefly, the extraction solvent (acetonitrile/water/acetic acid; 79/20/1) was added to the dried supernatant extract and after shaking and centrifugation, the extract was injected into the LC–MS/MS equipped with a TurboV electrospray ionization (ESI) source. The Phenomenex C18-column (150 × 4.6 mm, 5 µm) fitted with a C18 security guard cartridge (4 × 3 mm) was used to separate the compounds. The mobile phase consisted of methanol/water/acetic acid with the ratio of 10/89/1 (v/v/v) for eluent A and 92/2/1 (v/v/v) for eluent B. Both eluents contained 5 mM ammonium acetate.

Lactic acid and acetic acid were subsequently quantified using HPLC. The supernatant containing organic acids were diluted using 5 mmol/L sulphuric acid (H_2_SO_4_) and filtered using nylon syringe filters (0.22 µm pore size, Fisher) into HPLC vials. The separation and quantification of lactic and acetic acids were made using the HPLC Shimadzu UFLC XR, CTO-20A system consisting of a UV/VIS detector, a column oven, a degasser and an autosampler. The separation was done at 55 °C using a Rezex ROA-Organic Acid H^+^ (8%) column (150 × 7.8 mm) fitted with a guard column (security guard, 4 mm × 3 mm cartridge, Phenomenex, USA). The mobile phase was 5 mmol/L H_2_SO_4_. The flow rate, injection volume, detection wavelength and run time was 0.8 mL/min, 10 µl, 210 nm and 15 min, respectively. The retention time was at 5.0 and 5.9 min for L-lactic acid and acetic acid, respectively. A mixture of L-lactic acid (0.5–2.5 mg/mL) (R^2^ = 0.9999) and acetic acid standard (4–20 µg/mL) (R^2^ = 0.9999) was prepared by dissolving a stock solution in 5 mmol/L H_2_SO_4_. The Limit of Detection (LOD) for L-lactic acid and acetic acid were 0.03 mg/mL and 0.18 µg/mL, respectively. The Limit of Quantification (LOQ) was 0.1 mg/mL for L-lactic acid and 0.62 µg/mL for acetic acid. The mMRS broth was analysed as a control.

### Comparison of the inhibitory effect of between *Lp *HT-W104-B1 supernatant and selected compounds identified in the supernatant for control of *T. rubrum*

Sterile dried *Lp* HT-W104-B1 supernatant, L-lactic acid and acetic acid, were dissolved in sterile distilled water. The concentration and the pH of the L-lactic acid and acetic acid used in this study was the same as the concentration calculated as 20 mg/mL and the pH of the supernatant (pH 3.3). These antifungal compounds (100 µL each) were then mixed with keratin agar (approx. 52 °C) and poured into Petri plates (∅ 53 mm). The final concentration of supernatant, L-lactic acid and acetic acid were 20 mg/mL, 6.6 mg/mL and 2.1 µg/mL, respectively. The antifungal activity against *T. rubrum* was examined using the agar spot assay and incubated at 30 °C for 21 days.

### Agar spot assays

The agar medium (approximate 52 °C) was mixed with the supernatant and poured into Petri plates (53 mm ∅). After the agar media had solidified, 5 µL of spore suspension (2–5 × 10^6^ spores/mL) was carefully placed in the centre of the agar medium. The spore suspension was left to dry before being incubated at 30 °C. The fungal colony diameters were measured in two directions at right angles to each other and the colony area was calculated and compared with that of the control.

### Statistical analysis

The data was analysed for whether they followed a normal distribution using the Shapiro–Wilk W Test. For data with a normal distribution, the general influence of the supernatant on *T. rubrum* growth was checked using ANOVA. The Kruskal Wallis Test was used for non-normally distributed data. Students’ *t* test was used to compare the means for each treatment for normally distributed data. The Wilcoxon method was used for non-normally distributed datasets. p < 0.05 was used as the test for significant differences between treatments. JMP Pro (SAS Institute Inc., Cary, North Carolina, USA) was used for the analyses. All experiments were carried out with at least three replicates per treatment and repeated once.

## Results

### Screening of the LABs for efficacy against *T. rubrum* and *T. interdigitale*

**Fig. 1:**
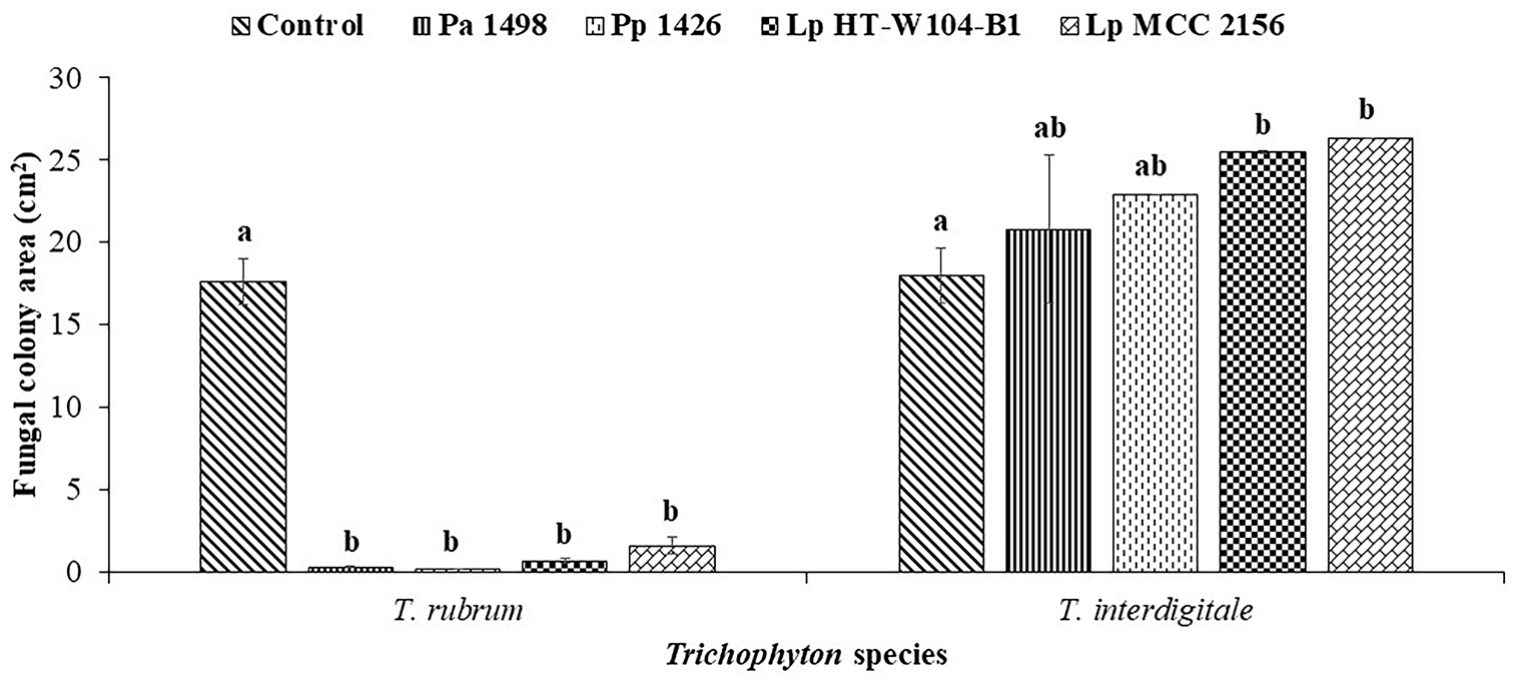
Effect of interaction between LABs and dermatophyte fungi on the fungal colony area. Bars indicate standard deviation of the mean. Different letter indicates significant difference (p < 0.05) within the treatment by Student’s *t* test. *Pa* 1498: *P. acidilactici* strain 1498, *Pp* 1426: *P. pentosaceus* strain 1426, *Lp* HT-W104-B1: *L. plantarum* strain HT-W104-B1, *Lp* MCC 2156: *L. plantarum* strain MCC 2156

Screening showed that of all the LABs, four strains appeared to be very effective against *T. rubrum* (*Lp* HT-W104-B1, *Lp* MCC 2156, *Pp* 1426 and *Pa* 1498; Fig. 1). There were no effects of any of the LABs on growth of the other dermatophyte, *T. interdigitale.* The reduction of *T. rubrum* colony area was significant (p < 0.05) when compared with the control, although there was no differences between the efficacy of the four LABs. Table 1 shows the interaction scores and Index of Dominance (I_D_) for the four LAB strains and the *Trychophyton* species. Interaction scores provide information on the type of macroscopic interaction between the competing strains/species. The I_D_ is the numerical sum of the interaction scores for each competing species to get an overall view of the best competitor, regardless of whether this is due to metabolite production or direct colony interactions. The four LABs that were most effective against *T. rubrum* all inhibited growth at a distance. In contrast, the LABs were uncompetitive against *T. interdigitale* where mutual intermingling occurred. Overall, all the LAB strains had similar I_D_ totals. Thus, studies were subsequently only focused on *T. rubrum*.

**Table 1:** Effect of interaction between LAB strains and *Trichophyton* species on the numerical interaction scores and total Index of Dominance (I_D_)

Bacterial strains	Interaction scores		Index of dominance (I_D_)
	*T. rubrum*	*T. interdigitale*	
*L. plantarum* HT-W104-B1	5:0	1:1	6:1
*L. plantarum* MCC 2156	5:0	1:1	6:1
*P. acidilactici* 1498	5:0	1:1	6:1
*P. pentosaceus* 1426	5:0	1:1	6:1

### Effect of supernatant of the four LAB strains on *T. rubrum* growth

**Fig. 2:**
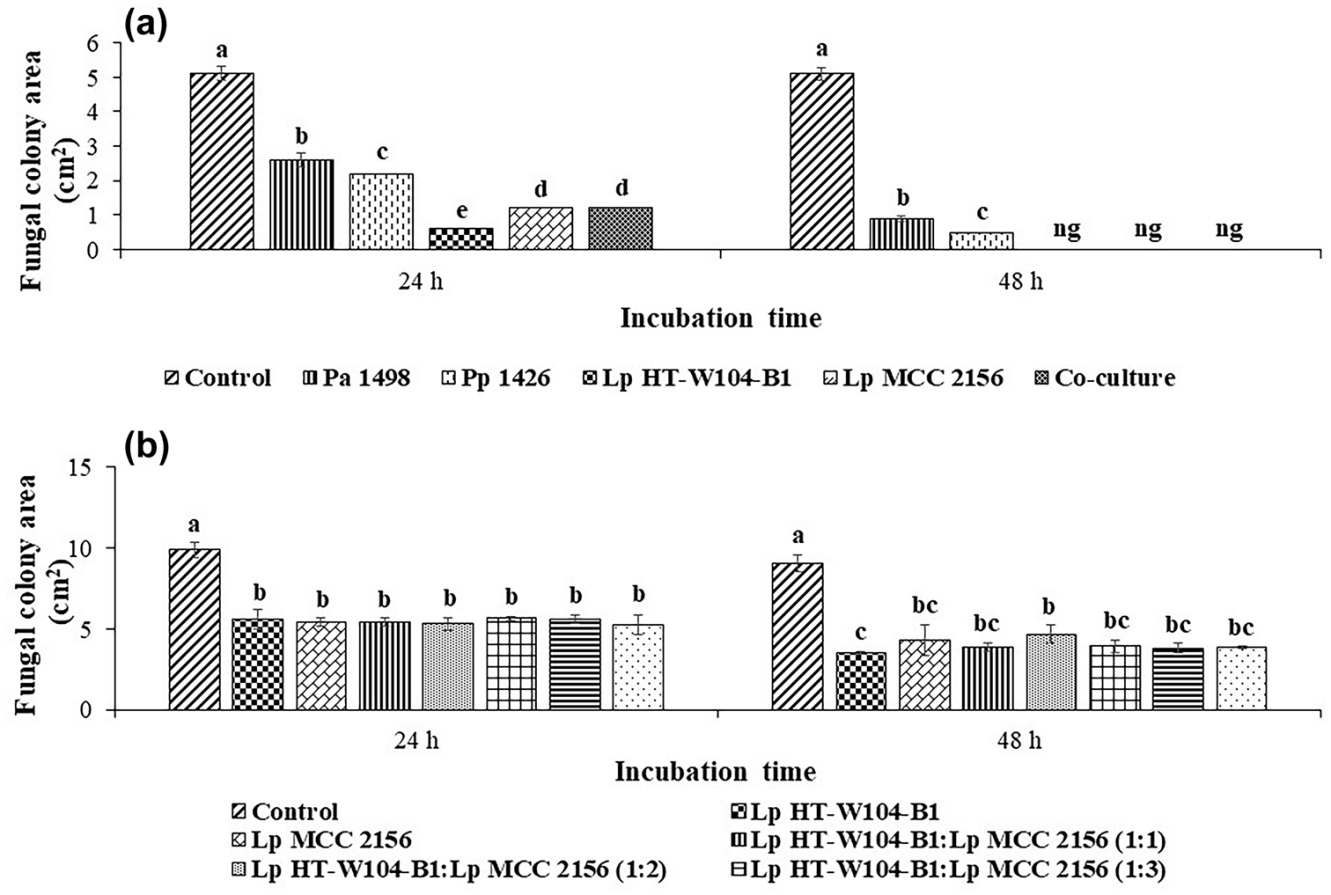
Effect of **a** *Pa* 1498, *Pp* 1426, *Lp* HT-W104-B1, *Lp* MCC 2156 and a mixture of four strains and **b** different combinations of *Lp* HT-W104-B1 and *Lp* MCC 2156 on antifungal activity against *T. rubrum* grown on SDA. Bars indicate standard deviation of the mean. Different letter indicates significant different (p < 0.05) within treatment using the Student’s *t* test. ng: no growth. *Pa* 1498: *P. acidilactici* strain 1498, *Pp* 1426: *P. pentosaceus* strain 1426, *Lp* HT-W104-B1: *L. plantarum* strain HT-W104-B1, *Lp* MCC 2156: *L*. *plantarum* strain MCC 2156

Figure 2a shows the effect of the supernatant of the four LAB strains on the fungal colony area after 24 and 48 h incubation at 30 °C. This was significantly reduced (p < 0.05) after 24 and 48 h when compared to the control. There were some differences between the efficacy of the LAB strains. The highest reduction was by *Lp* HT-W104-B1 followed by *Lp* MCC 2156 and a mixture of the four LABs including the two *Pediococcus* strains (*Pp* 1426 and *Pa* 1498). The inhibition was more pronounced after 48 h, with complete inhibition by *Lp* HT-W104-B1, *Lp* MCC 2156 and a mixture of the four LAB strains.

### Efficacy of *Lp* HT-W104-B1 and *Lp* MCC 2156 and a combination of the two strains for control of growth of *T. rubrum*

Figure 2b compares the efficacy of *Lp* HT-W104-B1 and *Lp* MCC 2156 and a mixture of these two strains on *T. rubrum* growth. There was a significant reduction (p < 0.05) of the *T. rubrum* colony area when compared with the control. However, the antifungal activity of *Lp* HT-W104-B1 and *Lp* MCC 2156 and combinations of these two strains was similar after both 24 h and 48 h. However, the supernatant of *Lp* HT-W104-B1 gave the best inhibition after 48 h.

### Effect of incubation time on the temporal kinetics of cell concentration of *Lp* HT-W104-B1 on control of *T. rubr*um growth

Table 2 shows the temporal changes in growth of *Lp* HT-W104-B1, the pH of the supernatant, organic acid production and effects on the colony area of *T. rubrum* over 60 h incubation. The growth of this LAB strain was rapid over the first 24 h, with the CFUs increasing significantly from 0.7 log_10_ CFUs/mL after 4 h to 3.7 log_10_ CFUs/mL after 48 h. This was followed by a plateau and then a slight decrease after 60 h incubation. The pH of the supernatant was significantly reduced (p < 0.05) over the incubation period. A significant reduction in *T. rubrum* colony area (p < 0.05) by the cell free supernatant of this LAB occurred after 8 h until the end of the experiment. The production of lactic acid increased slowly in the first 8 h and then significantly (p < 0.05) after 12 h. This was maintained for 24 h and then increased significantly (p < 0.05) after 36 h. Acetic acid was produced later than lactic acid, and first detected after 8 h and significantly increased after 24 h. A second significant biosynthesis period occurred after 36 h.

**Table 2:** Growth kinetic of *Lp* HT-W104-B1, pH of supernatant, *T. rubrum* colony area, lactic and acetic acid production during 60 h incubation at 30 °C

Incubation time (min)	pH of supernatant	Bacterial growth (log_10_ CFU ml^−1^)	Fungal colony area (cm^2^)	Organic acid (mg ml^−1^)	
				Lactic acid	Acetic acid
0	6.2 ± 0.0^a^	6.7 ± 0.02^f^	7.3 ± 0.72^ab^	0 ± 0^d^	0 ± 0^d^
2	5.8 ± 0.0^b^	7.0 ± 0.02^f^	7.4 ± 0.11^a^	0.01 ± 0.005^d^	0 ± 0^d^
4	5.7 ± 0.01^c^	7.4 ± 0.05^e^	7.5 ± 0.43^a^	0.032 ± 0.016^d^	0 ± 0^d^
8	4.9 ± 0.04^d^	8.7 ± 0.06^d^	7.5 ± 0.36^a^	0.18 ± 0.002^d^	0 ± 0^d^
12	4.0 ± 0.02^e^	9.1 ± 0.02^c^	6.6 ± 0.01^b^	0.41 ± 0.014^c^	0.07 ± 0.002^c^
24	3.5 ± 0.03^f^	10.0 ± 0.09^b^	3.6 ± 0.17^c^	1.22 ± 0.083^b^	0.09 ± 0.005^b^
36	3.4 ± 0.04^g^	10.1 ± 0.03^ab^	3.2 ± 0.78^c^	1.24 ± 0.274^b^	0.08 ± 0.015^bc^
48	3.2 ± 0.02^h^	10.4 ± 0.61^a^	1.3 ± 0.37^d^	1.98 ± 0.141^a^	0.10 ± 0.006^a^
60	3.2 ± 0.01^i^	9.9 ± 0.13^b^	1.0 ± 0.10^d^	2.00 ± 0.069^a^	0.11 ± 0.012^a^

Data are means of triplicates ± SD. Different letters indicate significant differences (p < 0.05) using the Student’s *t* test

### Effect of supernatant treatments of the LAB *Lp* HT-W104-B1 on control of *T. rubrum* growth

**Fig. 3:**
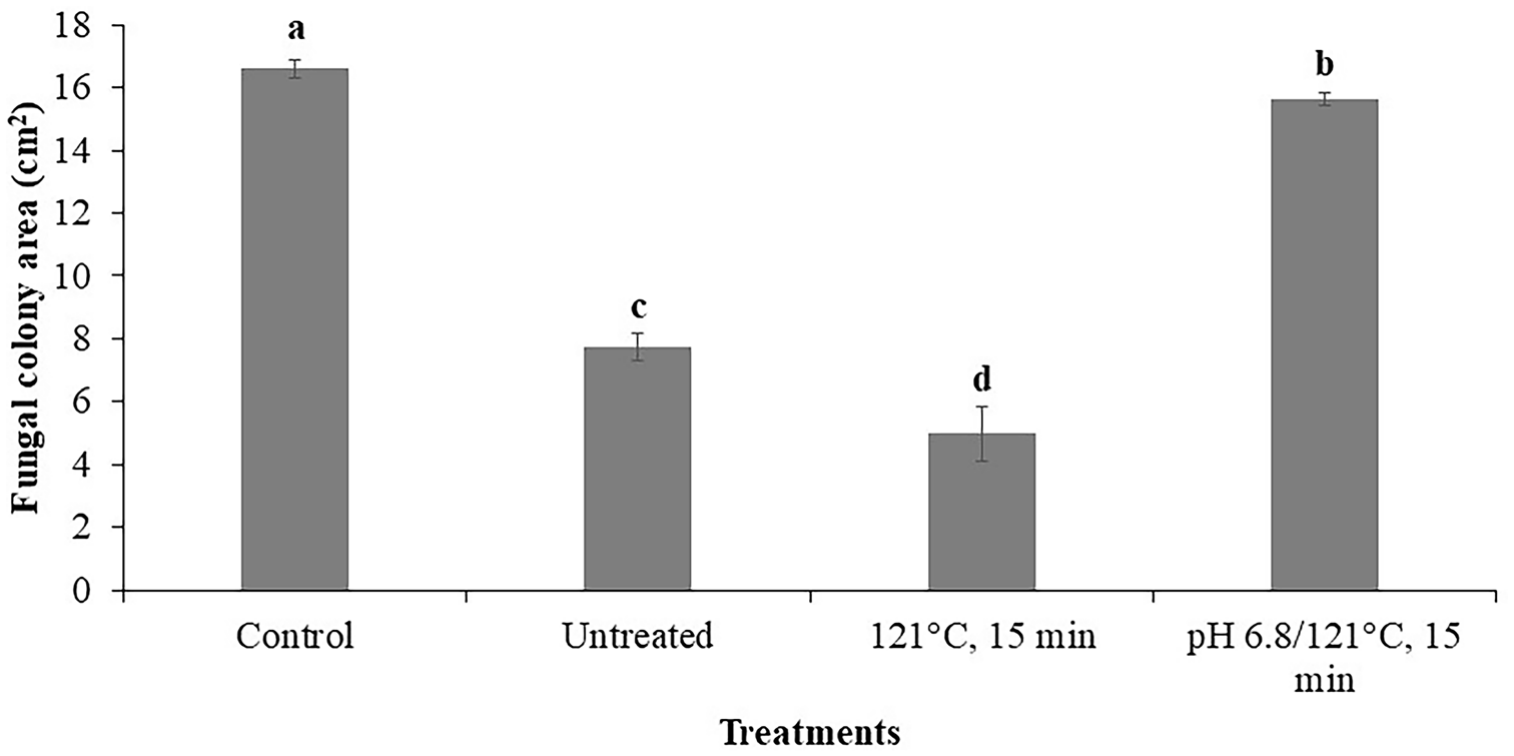
Antifungal activity of *Lp* HT-W104-B1 supernatant after heat treatment with/without modification to pH 6.8. The unmodified pH of supernatant was 3.3. Bars indicate standard deviation of the mean. Different letters indicate significant differences (p < 0.05) using the Student’s *t* test

Figure 3 shows the effect of heat treatment and modification of the pH of the supernatant of this LAB on the efficacy against *T. rubrum* growth. There was a significant reduction (p < 0.05) in *T. rubrum* colony area in the heated supernatant treatment (at 121 °C, 15 min) when compared to the control and the untreated supernatant. However, when the pH was modified to 6.8 + heat treatment, the *T. rubrum* colony area was significantly increased (p < 0.05) compared to the untreated supernatant alone. However, it was significantly reduced (p < 0.05) when compared to the control.

### Isolation of the anti-fungal compounds from LAB *Lp* HT-W104-B1 cell free supernatant

A total of six compounds secreted by *Lp* HT-W104-B1 were isolated and identified from the cell free supernatant (Table 3). The predominant components were two organic acids, L-lactic acid and acetic acid. Four minor compounds were also present.

**Table 3:** Concentration of organic acids and other metabolites in cell free supernatants of *Lp* HT-W104-B1 grown in mMRS broth for 48 h at 30 °C

Antifungal metabolites	Concentration (mg g^−1^ cell dry weight)
Organic acid	
L-lactic acid	19,113.0 ± 66.2
Acetic acid	2.2 ± 0.1
Other metabolites	
Cyclo(L-Pro-L-Val)	0.0551
Tryptophol	0.0005
Culmorin	0.0003
Phenopyrrozin	0.0001

### Efficacy of the LAB *Lp* HT-W104-B1 supernatant against *T. rubrum* when grown on keratin-based matrices

The efficacy of the different concentrations of the mixture of supernatant compounds against *T. rubrum* was quantified. The minimum inhibitory concentration (MIC), concentration for inhibition of 50% growth (IC_50_) and minimum fungicidal concentration (MFC) of the supernatant of this LAB were found to be 20 mg/mL, 14 mg/mL and 30 mg/mL, respectively.

**Fig. 4:**
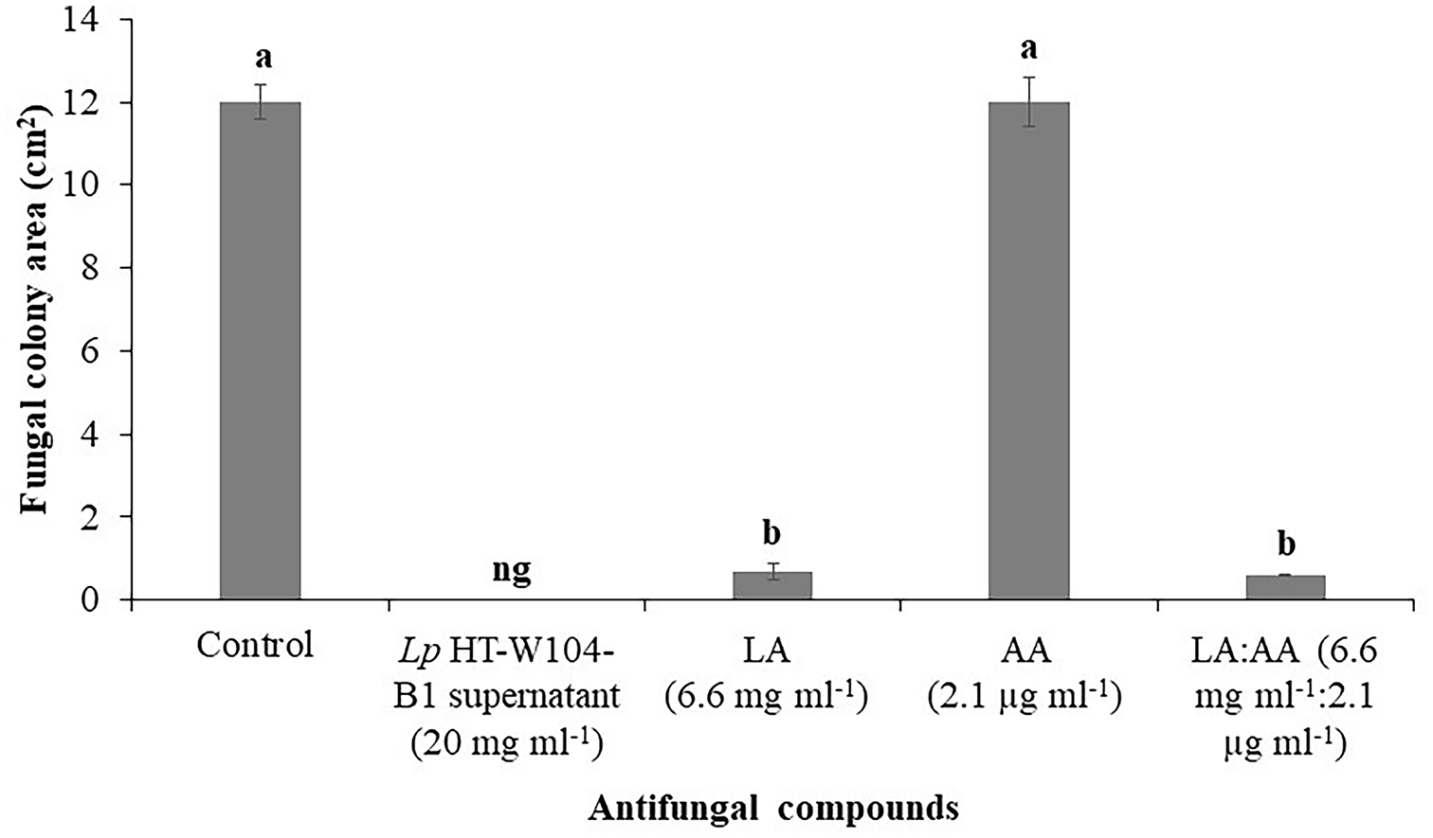
Efficacy of *Lp* HT-W104-B1 supernatant, L-lactic acid and acetic acid for control of growth of *T. rubrum*. Bars indicate standard deviation of the mean. Different letter indicates significant differences (p < 0.05) using the Students’ *t* test. LA: L-Lactic acid, AA: Acetic acid, ng: no growth

The efficacy of the mixture of compounds in the LAB supernatant was compared with the two individual compounds (L-lactic acid and acetic acid) to control growth of *T. rubrum*. Figure 4 shows that with the natural mixture of compounds from the LAB supernatant there was complete inhibition of growth of *T. rubrum.* L-lactic acid alone, or in combination with acetic acid, also resulted in a significant reduction (p < 0.05) in growth of *T. rubrum* when compared to the control, or to the acetic acid treatment alone. However, the use of acetic acid alone was ineffective with growth similar to the control treatment.

## Discussion

This is the first detailed screening of a wide range of LABs isolated from Malaysian fermented foods and their metabolites for efficacy against important human pathogens such as dermatophytes. Overall, of the 66 LABs examined, only two strains each of *Lactobacillus* and *Pediococcus* had strong anti-fungal activity against one of two dermatophytes tested. This suggests that the anti-fungal activity is not only dependent on the individual LAB strain (Cheong et al. 2014) but also on the target fungal species (Gupta and Srivastava, 2014). Previously, Magnusson et al. (2003) and Cheong et al. (2014) found that strains of *Lactobacillus* and *Pediococcus* were more effective in controlling target fungal pathogens.

Interestingly, the four LAB strains are more effective against *T. rubrum* than *T. interdigitale* although generally the growth of *T. rubrum* is more difficult to control than *T. interdigitale* (Salehi et al. 2018; Intra et al. 2019). Compared to *T. interdigitale*, *T. rubrum* is able to produce a more compact and denser biofilm, which is crucial for protection from anti-fungal agents and for its survival (Costa-Orlandi et al. 2014). According to our results, *T. interdigitale* was not susceptible to the metabolites secreted by the LAB strains examined. Previously, it was reported that the susceptibility of *T. interdigitale* strains from different hospitals sources were variably affected by anti-fungal drugs (Intra et al. 2019). Some of these strains even showed resistance to multiple drugs such as terbinafine, fluconazole, variconazole and itraconazole that are commonly used clinically, to treat dermatophytic infections (Singh et al. 2018). Studies at a molecular level have related this resistance to point mutations of one of the four amino acids located at different positions (Leu393, Phe397, Phe415 and His440) in the squalene epoxidase (*SQLE*) gene (Salehi et al. 2018; Singh et al. 2018).

It was noted that the control obtained with *L. plantarum* strains was more effective than that with the *Pediococcus* strains. This inhibition could partially have been due to the acidification of the supernatant of the *L. plantarum* strains. For example, the final pH values of *Lp* HT-W104-B1, *Lp* MCC 2156, *Pp* 1498 and *Pa* 1426 were 3.14, 3.20, 3.33 and 3.43, respectively. Interestingly, co-cultivation of the four LABs in this study also significantly enhanced the inhibition when compared to the individual *Pediococcus* strains, but not when compared with a single *L. plantarum* strain. Overall, treatment with the LAB *Lp* HT-W104-B1 alone was more effective than when co-cultivated with the other strains. Use of the co-cultivation of the two *L. plantarum* strains did not increase the efficacy in controlling growth of *T. rubrum* than an individual one. Previous studies have suggested that co-cultivation can provide some benefits for inhibition of growth of both bacterial or fungal pathogens when using co-cultures of bacteria + bacteria (Rojo-Bezares et al. 2007), bacteria + fungal pathogen (Esmaeilishirazifard et al. 2018) or fungus + fungal pathogen (Azzollini et al. 2018) co-cultivation. It has been suggested that the advantage of such co-cultivation may be due to enhanced secretion of antagonistic substances or the induction of new stress-related compounds such as antibiotics or secondary metabolites with anti-microbial and cytotoxic activity (Marmann et al. 2014). In addition, the induction of bacteriocin activity by LABs was more pronounced when the mixture included living cells or partially heat-denatured cells (Rojo-Bezares et al. 2007). However, co-cultivation has not always resulted in a significant enhancement in antimicrobial activity of a LAB strain. Tirloni et al. (2014) found that the antibacterial activity of the *L. animalis* SB310 and *L. paracasei* subsp *paracasei* SB137 mixture varied depending on the anti-microbial efficacy of the individual LAB strain and the ratio of the mixture.

In the present study co-cultivation of both *L. plantarum* strains did not enhance the control of *T. rubrum* growth. Thus, the studies were focused on one specific LAB strain, *Lp* HT-W104-B1. This gave the best inhibitory activity when compared with the other three LAB strains tested. The best inhibitory effect of this strain was identified as during the late log phase, and this continued during the subsequent stationary growth phase. It was hypothesised that probably acids such as lactic and acetic acid secretion by this strain may be involved in this inhibitory activity because of the high concentrations produced during the log phase of growth. This was confirmed by quantification of the spectrum of metabolites produced by this strain of LAB was predominantly lactic and acetic acids with mixtures of minor compounds. Previous studies have also found that specific LAB strains produced individual or mixtures of aliphatic acids that contribute to efficacy. Among these were lactic, acetic, propionic, formic, succinic, butyric, phenyllactic, citric, oxalic, malonic acids or mixtures of some of these (Crowley et al. 2013; Özcelik et al. 2016; Şehirli and Savdam, 2016; Valerio et al. 2016).

There has been significant interest in a better understanding of the mechanism of action of potential anti-fungal compounds against human pathogens including dermatophytes (Martinez-Rossi et al. 2018). Observations have included morphological alteration of hyphae and conidia and ultrastructural changes in the fungal cells, especially in permeability of the cell wall/membrane (Ghahfarokhi et al. 2004; Ahmad Khan and Ahmad 2011; De Oliveira Pereira et al. 2013; Avanco et al. 2017). In addition, the release of intracellular material, primarily nucleotides, as an indication of leakage from the fungal cells has been quantified (De Oliveira Pereira et al. 2013). However, sub-optimal concentrations of fungicides may promote compensatory stress responses, with the over-expression of genes involved in cellular detoxification, drug efflux, and signalling pathways amongst the mechanisms that may contribute to tolerance to antifungal compounds in dermatophytes (Martinez-Rossi et al. 2018). However, the effects of antifungal compounds on hydrolytic enzyme production by *T. rubrum* and other dermotophytes may also be important.

Hydrolytic enzymes are critical for the life and infection cycle of fungal pathogens including dermatophytes such as *T. rubrum*. Studies by Mohd Danial (2019) showed that on a keratin based-medium the mixed naturally produced compounds significantly decreased the temporal production of alkaline phosphatase, esterase and N-acetyl-β-glucosaminidase. Interestingly, keratinase activity was increased with 15 mg/mL of the mixture of compounds from the best *Lactobacillus plantarum* species. This is a key enzyme for infection of keratin rich matrices and it may be involved in both growth and sporulation. Thus under chemical imposed stress, growth may be reduced but sporulation is increased and could be related to the enhanced keratinase activity (Sharma et al. 2017; Mohm Danial 2019).

In this study, pH neutralization and heat treatment (121 °C/15 min) indicated that the supernatant was active at low pH (3.33; original pH), and lost this activity after neutralization. Interestingly, heat treatment improved the control efficacy achieved. This may have resulted in some denaturing or alteration of the primary configuration of some of the compounds secreted by the *L. plantarum* strain, perhaps working in an additive or synergistic way with the lactic or acetic acid against *T. rubrum* (Qian et al. 2017). L-lactic acid was found to be the most abundant organic acid produced by the *Lp* HT-W104-B1 strain, in significantly higher amounts than acetic acid. It was notable that the natural mixture of compounds in the supernatant of this LAB strain was more inhibitory against *T. rubrum* than the pure compounds alone (lactic or acetic acid). However, lactic acid was a major compound responsible for the inhibitory effects observed. The efficacy of weak acids to control the growth of microbial pathogens was reported previously (Shokri 2011; Sharma and Srivastava 2014) and it was more significant when the pH was < pKa value (Kundukad et al. 2020). Although the pH of both lactic and acetic acid in our study was adjusted to pH 3.33 (< pKa), no activity was observed for acetic acid. This was in contrast to Kuwaki et al. (2002) who found better control of tineal fungal growth by acetic than lactic acid. This discrepancy could be due to the very low concentration of acetic acid applied in our study that was not high enough to inhibit the growth of the strain of *T. rubrum* used. It has been postulated that the undissociated organic acid with a low pH will diffuse through the membrane of the targeted organism. The high pH of cytoplasm favours the dissociation of organic acids that releases protons and acidifies the cytoplasm. This eventually slows down or completely inhibits the cell growth because significant energy is expended in neutralizing the internal pH (Schillinger and Villarreal, 2010; Scorzoni et al. 2018).

In summary, this study has identified that some LAB strains isolated from Malaysian fermented foods could be excellent sources of anti-fungal compounds that could inhibit specific medically important skin disease pathogens such as *T. rubrum.* Further in situ tests are now required for examining control when compared to existing treatments. Natural mixtures of aliphatic acids and other compounds could represent effective sources for the development of alternative drug leads for the control of fungal pathogens that are developing resistance to existing fungicides.
